# Strengthening Somalia’s health system: pathways to achieving International Health Regulations core capacities at points of entry by 2025

**DOI:** 10.1186/s41182-025-00836-z

**Published:** 2025-11-10

**Authors:** Saadaq Adan Hussein, Marian Muse Osman, Yahye Sheikh Abdulle Hassan, Abdirahman Aden Hussein, Rage Adem, Ayan Nur Ali, Mohamed Farah Yusuf, Abubakar Nor Farah Shurie, Abdinur Adan Hussein, Omar Mohamed Mohamud, Abdullahi Mohamed Mohamud, Abdirahman Moallim Ibrahim, AbdulJalil Abdullahi Ali, Chukwuma David Umeokonkwo

**Affiliations:** 1 Department of the School of Postgraduate Studies, Benadir University, Hodan Benadir, Mogadishu, Somalia; 2 Department of Social and Human Capital Development Pillar, Office of the Prime Minister, Mogadishu, Somalia; 3 Department of Research and Policy Development, SOR Institute: Somalia Social Research, Mogadishu, Somalia; 4 Benadir Institute for Research and Development, Benadir University, Mogadishu, Somalia; 5 Department of Research, Somali National Institute of Health, Mogadishu, Somalia; 6 Department of Medicine and Surgery, Jamhuriya University of Science and Technology, Mogadishu, Somalia; 7 Department Innovation Hub, Benadir University, Mogadishu, Somalia; 8 Department of Hemodialysis, Mogadishu Somali Türkiye Training and Research Hospital, Mogadishu, Somalia; 9 Tayo Institute for Research and Development, Mogadishu, Somalia; 10 Department of Civil Engineering, Amoud University, Borama, Somalia; 11 Department Faculty of Medicine and Surgery, Jazeera University, Mogadishu, Somalia; 12 African Field Epidemiology Network, Kampala, Uganda

**Keywords:** Somalia, System International, Regulations, Health, Security, Disease, Surveillance, Governance, Infrastructure

## Abstract

**Introduction:**

The *International Health Regulations (2005)* (IHR) mandate global health security through core capacities, yet Somalia’s 48 Points of Entry (PoEs), including airports, seaports, 12 domestic airports, 6 international airports, and land borders, lack essential infrastructure, staffing, and surveillance. Somalia allocates only 1.3% of the government budget to health, far below the Abuja Declaration target of 15%, with 75% of domestic airports lacking medical staff. The study aimed to enhance Somalia’s PoEs control by analyzing existing systems, identifying gaps, and comparing countries and pathway resilience strategies.

**Methods:**

Following the Preferred Reporting Items for Narrative Reviews by SANRA guidelines, this Narrative review analyzed 118 data. The data for this study were collected from multiple sources: peer-reviewed articles, government reports, and datasets. Searches across PubMed, Scopus, and Google Scholar used terms including (International OR regulation* OR “international health regulation*”) AND (“point* of entry*” OR surveillance) AND (response OR Somalia OR “horn of Africa” OR “core capacity*”). Data were coded in NVivo 12 using a hybrid approach of deductive coding mapped to WHO IHR (2005) PoE domains, and data were thematically analyzed across five domains.

**Results:**

For health system gaps, Somalia’s IHR compliance score (31 out of 100) reflects weak surveillance, workforce shortages (4.45 health workers per 1000 people), and fragmented governance. For PoEs deficiencies, 63% of sea ports lack screening measures; only 50% of international airports meet basic health security standards. For regional comparisons, Somalia trails Kenya (80% IHR capacity), Ethiopia (75% surveillance), and Rwanda (72% lab capacity) in preparedness. For key challenges, political instability disrupts coordination,

**Conclusion:**

Somalia’s progress in meeting IHR core capacities at Points of Entry (PoEs) by 2025 is critical for enhancing national resilience, global health security, and major challenges. Addressing these challenges requires significant investments in PoEs to achieve measurable outcomes.

## Introduction

The *International Health Regulations (2005)*, IHR (2005) for short, is a legally binding instrument for 196 countries, including all World Health Organization (WHO) Member States, aimed at ensuring global health security defining their rights and obligations in managing public health events that may cross borders [1, 2]. Globalization, urbanization, and climate change significantly contribute to the spread of infectious diseases and health security threats by influencing the survival of microorganisms and altering climatic and nutritional conditions in human and animal hosts [3, 4]. The WHO mandates capacities to prevent, detect, and respond to international health emergencies, with the responsibility for implementing IHR at the national level and State Parties must achieve 13 core-capacity areas by set deadlines (Table 3) [5–7].

The IHR is a crucial global framework for public health security, requiring countries to develop core capacities such as legislation, coordination, zoonotic events, food safety, laboratory surveillance, human resources, the national health emergency framework, health service provision, risk communication, Points of Entry (PoEs), chemical events, and radiation emergencies [8, 9]. High-volume traffic at PoEs, including airports, ports, and border crossings, facilitates the international spread of diseases through travelers, vehicles, and goods [10]. PoEs safeguard global health security through routine checks, enhanced screening, and rapid testing to detect, assess, report, and respond to public-health events and manage travel- and trade-related risks, while nationwide core capacities build, strengthen, and maintain surveillance and response functions to detect, assess, notify, and respond at the local/primary, intermediate, and national levels [11–15].

**Fig. 1:**
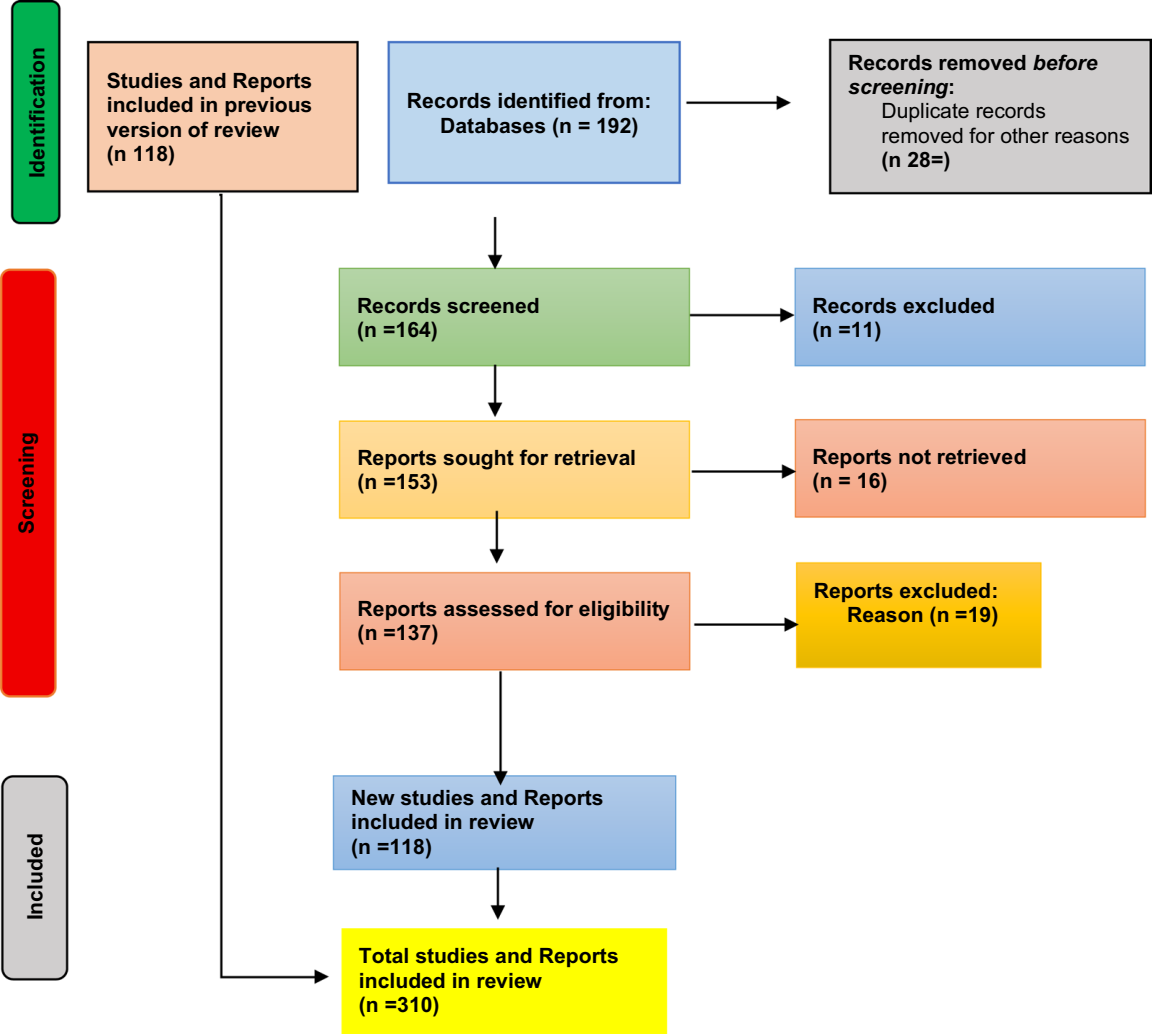
Organizational structure of the Ministry of Health related to portal health [21]. *Source*: Organogram, Ministry of Health, Somalia

Somalia has 48 Points of Entry (PoEs), including 12 domestic airports, 6 international airports, 22 land crossings, and 8 sea crossings [16]. These PoEs play vital roles in maintaining national and global health security [17]. However, Somalia faces health challenges at PoEs, especially domestic airports, where 69% lack medical staff and 73% highlight critical preparedness gaps [18]. The nation’s 20% capacity indicates a limited ability to conduct IPC or decontamination [19], with overall compliance measures among healthcare workers at 58.3%, including breaks with PPE at 55.9%, hand hygiene at 55.4%, and other IPC practices at 52.0% [20]. Somalia’s Ministry of Health, through the National Institute of Health Director, oversees a structured port health system to ensure IHR compliance and manage cross-border health threats (Fig. 1) [21]. Somalia’s health system struggles with IHR implementation due to weak infrastructure, instability, aid reliance [22], outbreaks, and personnel shortages [23, 24]. Somalia’s IHR capacity is weak [25]. Overall capacity is 35/100; key scores—Laboratory 70%, Surveillance 40%, National health emergency framework 40%, Risk communication 33%, Points of entry 27% [19]. Capacities were low across pillars—Prevent 1.2/5, Detect 1.9/5, Respond 1.9/5 (averaged from indicator scores) [26]. Somalia’s IHR compliance and health security can improve with better governance, capacity-building, and stronger infection prevention at PoEs [27, 28]. This study aimed to help enhance Somalia’s PoEs control through analyzing existing systems, identifying gaps, and comparing countries and pathway resilience strategies.

## Materials and methods

### Study design

**Fig. 2:**
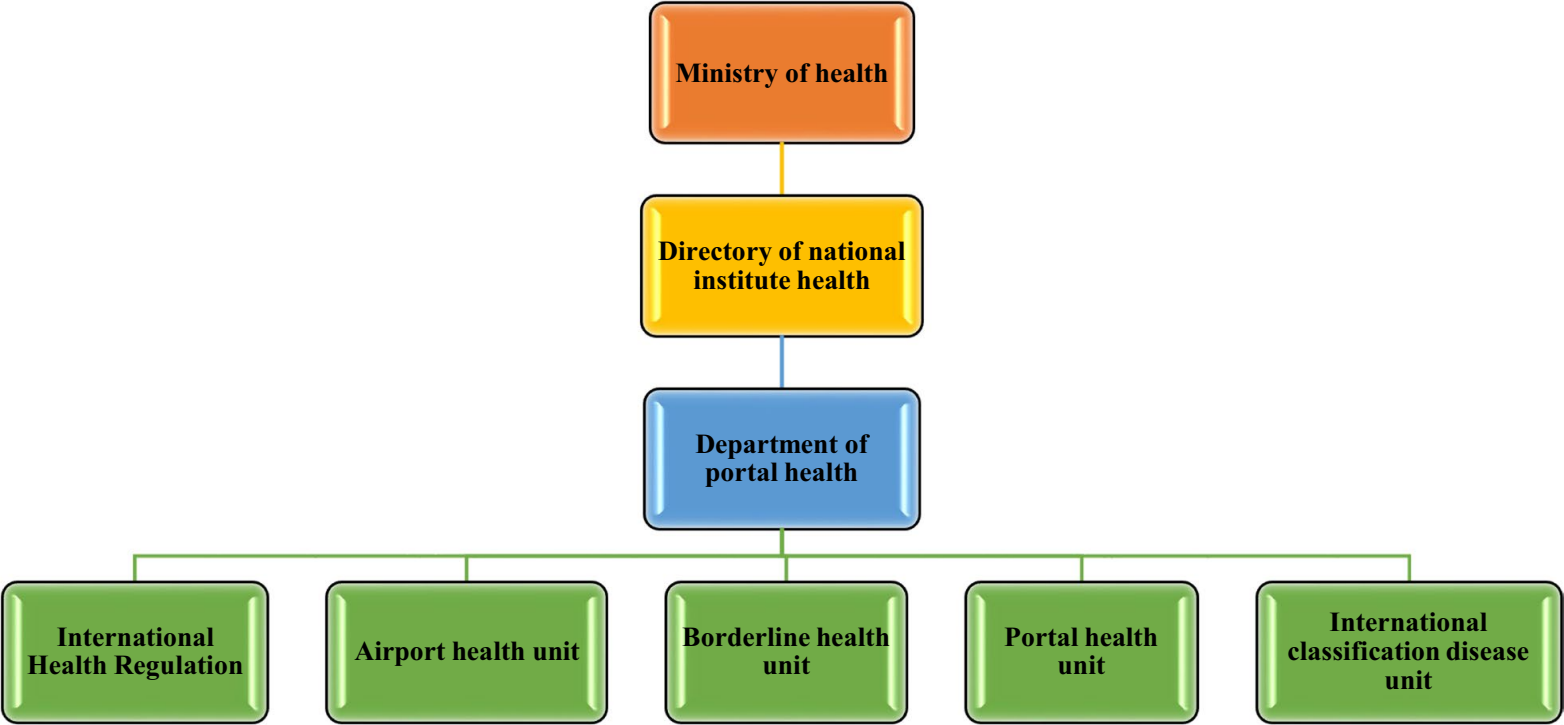
SANRA (scale for the assessment of narrative review articles) [29]

This study used a narrative review guided by SANRA (Scale for the Assessment of Narrative Review Articles) (Fig. 2) [29] to assess IHR core capacities at Somalia’s PoEs. Identifying IHR gaps at PoEs offers broader insights into Somalia’s health system performance.

### Data collection strategy and procedure

Searches were conducted October 2024–April 2025, with a final search on May 25, 2025; inclusion was limited to English. Data for this study were collected from multiple sources, including government and NGO reports, grey literature, and datasets from international and local organizations, selected based on relevance to themes such as IHR, PoEs, and core capacities in Somalia. For peer-reviewed articles, a narrative search was conducted across databases such as PubMed, Web of Science, Scopus, and Google Scholar, on the search terms (International OR regulation* OR “international health regulation*”) AND (“point* of entry*” OR surveillance) AND (response OR somalia OR “horn of Africa” OR “core capacity*”) AND (strengthen* OR “security core”)). Other data sources of grey literature retrieval. We sourced Ministry of Health publications, health facility reports, and national surveillance data—including official reports, operational datasets, and policy/guideline documents—relevant to surveillance, points of entry, response, and core capacity strengthening. Documents were identified via official MoH portals/archives, facility HMIS/DHIS2 repositories, and IDSR/EWARN bulletins. We included current, directly relevant materials and excluded duplicates/superseded versions; extracted issuing body, date, scope, and key metrics (e.g., completeness, timeliness, case counts, response); and appraised credibility by source authority, recency, and concordance with routine statistics.

### Inclusion and exclusion criteria

#### Inclusion criteria

Empirical studies on Somalia’s PoE relevant IHR (2005) capacities—quantitative (cross sectional/cohort/case control/time series), qualitative (KIIs/FGDs/document analysis), mixed methods, and official assessments (SPAR/JEE/IOM DTM).

Publication types and language: peer-reviewed articles/conference papers and credible grey literature (MoH/WHO/UN/Africa CDC/Health Cluster reports, IDSR/EWARN bulletins, national policies/SOPs/guidelines); English only.

#### Exclusion criteria

Studies not related to Somalia or IHR PoEs’ core capacities, or lacking sufficient methodological details.

### Thematic analysis

Data were coded in NVivo 12 using a hybrid approach in deductive coding mapped to WHO IHR (2005) PoE domains, complemented by inductive open codes. Two reviewers coded independently; disagreements were resolved by consensus with third-reviewer arbitration. Intercoder reliability was assessed on a 20% double-coded sample. Findings are organized into five themes: (1) current IHR core capacities in Somalia; (2) global health system ranking; (3) challenges to PoEs’ capacity; (4) comparisons with other African countries; and (5) pathways to achieve core capacities by 2025. Of 310 identified articles, 192 were screened and 118 met the inclusion criteria.

## Results

### Current situation of the core capacities of IHR in the health system of Somalia

**Fig. 3:**
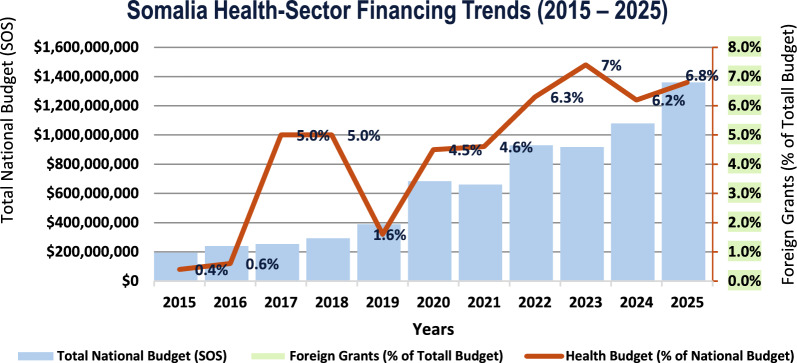
Somalia Health-Sector Financing Trends (2015–2025) [31]

Somalia’s total national budget rose from 0.15–0.20 billion in 2015 to 1.4–1.5 billion in 2025, while the health share increased from <1 to 7% in 2023 and 6.8% in 2025 (Fig. 3) [30, 31]. However, the government expenditure on health is far below the AU target of 15% [32], and its UHC Service Coverage Index is 27/100 versus a regional Sub-Saharan Africa average of 42.5 [33]. The country scores 31/100 on IHR compliance, indicating weak preparedness, surveillance, and response [34]. Under-five mortality stands at 106.1 per 1000 live births [35], and the maternal mortality ratio is 621 per 100,000 live births [36, 37]. Resource constraints, security issues, and weak infrastructure hinder IHR implementation [38, 39]. Food insecurity threatens 1.6 million children by 2025 and affects 4 million people between January and June 2024 [40, 41]. WHO urgent gaps in the implementation of the International Health Regulations (IHR) in Somalia have been identified, including insufficient resources, lack of trained personnel, and inadequate coordination among sectors [42]. Despite surveillance enhancements via IDSR implementation in Somalia, the progress in various thematic areas ranged from 15 to 78%, with the timeliness and completeness of health data reporting through the implementation of DHIS2 improving from a national average of 62% in 2016 to 91% in 2022 [43, 44]. International partners, notably WHO and UNICEF, are expanding vaccination and treatment access while addressing both communicable and non-communicable diseases [45–47]. Table 1 shows the current situation of Somalia’s health system.

**Table 1:** Current situation of Somalia’s health system

Indicator	Value or score	References
Health spending (% of government expenditure)	1.3% (Abuja Declaration target: 15%)	[57]
IHR compliance score	31	[34]
Under-five mortality rate (per 1000 live births)	106.1	[101–103]
Maternal mortality ratio (per 100,000 live births)	1044–1400	[23–27]
Children at risk of malnutrition (by 2025)	1.6 million	[104, 105]
Population in food insecurity (Jan 2024–June 2024)	4 million	[40]

*AU* African Union

### Global Health Security Index (GHS) indicators for Somalia

Somalia’s fragmented governance, limited resources, and weak infrastructure hinder IHR implementation [48]. Its 2021 GHS Index score is 31/100—among the lowest globally [49]. The decentralized health system in Somalia causes service inconsistencies, particularly in cross-border coordination. For example, varying levels of support and resources across regions lead to unequal healthcare access. Weak coordination between federal and state authorities hinders effective responses to health issues, such as disease outbreaks, in border areas [32]. Somalia’s health system has been severely weakened by decades of civil conflict, resulting in some of the lowest health indicators in the world [50]. Somalia’s PoEs face a significant screening deficit, including temperature checks, health questionnaires, and visual inspections, with 75% of domestic airports, 73% of land borders, 50% of international airports, and 63% of sea borders lacking the necessary screening measures to detect potential threats [51]. The country’s IHR suffer from insufficient screening, resources, and funding, which increase the risk of disease and limit the ability of crisis response [52]. In addition, cholera has been a persistent issue in Somalia since the 1990s, contributing to cross-border spread and further straining the healthcare system [53]. Somalia scores poorly on the Global Health Security Index, with an overall score reflecting weak healthcare access and infection control. It scores 1.0 in medical surge capacity, 21.9 in international norms, and zero in IHR reporting and cross-border health. The risk environment is rated at 23.6 due to instability and poor infrastructure. Prevention scores 11.4 (0 in biosecurity, 50 in immunization), detection/reporting 11.7 (limited labs and surveillance), and response 25.8 (0 in emergency operations, 37.5 in risk communication) [49].

### Challenges to achieving core capacities for PoEs in Somalia’s health systems

#### Political instability, security concerns, and restricted access

Political instability in Somalia severely undermines its health systems, particularly at PoEs [54]. Fragmented governance disrupts surveillance at airports, seaports, and land borders, reducing the ability to detect health threats such as infectious diseases [55]. Conflicts delay outbreak responses, divert resources from public health to security, and leave PoEs understaffed and lacking essential supplies like testing kits and protective equipment [56]. Cross-border movements in conflict zones heighten the risk of communicable diseases, while poor infrastructure and governance limit the capacity of the Ministry of Health to address these challenges. Insufficient health spending in Somalia, as a percentage of total government expenditure, exacerbates the structural weaknesses and service gaps [32, 57]. Poverty and insecurity further restrict people’s access to healthcare, especially in conflict-affected areas, perpetuating poor health outcomes [58, 59]. Addressing these issues requires prioritizing investments in health to strengthen resilience and economic growth [60, 61].

#### Inadequate funding and infrastructure

Inadequate funding and heavy reliance on inconsistent donor aid create vulnerabilities in Somalia’s health system, often causing interruptions in essential services [62]. Health facilities frequently lack supplies and equipment, resulting in substandard care and preventable diseases [63]. Low pay and poor conditions drive away skilled health workers, worsening shortages and services. Limited resources weaken surveillance, emergency response, and training, raising outbreak risks. These gaps harm health, reduce productivity, and slow economic growth. Sustainable domestic funding is essential [64, 65].

Poor infrastructure accelerates infectious disease spread and impedes emergency care and quality service delivery at PoEs [66, 67]. Inadequate facilities and equipment delay treatment and increase morbidity in rural regions [68]. Additionally, weak disease surveillance at PoEs allows infectious diseases to spread unchecked, further straining the country’s fragile health system [11, 69]. Weak surveillance at PoEs allows unchecked disease transmission, straining the health system further.

Decentralization in Somalia’s healthcare system, particularly at PoEs, has caused governance fragmentation, service disparities, and weak stakeholder coordination [70]. Regional autonomy in Somalia has caused inconsistent healthcare policies, unequal health financing, and a fragmented workforce, leading to disparities in service quality and access [71, 72]. The Federal Ministry of Health (FMoH) struggles with ineffective communication, unclear structures, and limited resources, leading to uncoordinated health policy implementation [64]. Fiscal inefficiencies, reliance on external aid, and fragmented funding further weaken infrastructure and service delivery [32, 73].

### Comparison of health systems at PoEs in different African countries

Somalia operates at only 33% IHR implementation capacity amid crises like drought and displacement [74], significantly lagging behind Kenya (80%) and Uganda (60%) due to its weak legal framework [25, 75, 76]. Somalia’s surveillance capacity is 30%, hindered by fragmented data practices, unlike Ethiopia (75%) and South Africa (80%) with effective systems. Strengthening Somalia’s system is essential for timely outbreak detection and response [25]. Somalia’s IDSR system, launched in 2020, improves disease surveillance, lab capacity, event-based monitoring, and rapid response [77]. Somalia’s health workforce capacity is 25%, far below South Africa (85%) and Tanzania (75%), limiting service delivery and public health response [25].

Somalia has fewer than 0.11 health workers per 1000 people [78], well below the Sustainable Development Goals (SDGs) target of 4.45 per 1000 people. This results in a shortfall of about 24,000 healthcare professionals needed to meet the minimum service levels [79]. Somalia’s lab capacity is 30%, far below Nigeria (75%) and Rwanda (72%), limiting accurate disease detection and response [25]. Despite Africa CDC support, lab systems require major upgrades [80]. Somalia’s cross-border health collaboration capacity is 25%, compared to Rwanda (80%) and Kenya (78%). Stronger partnerships are needed to manage regional disease threats [25]. Africa CDC’s framework supports cross-border surveillance [81]. Somalia scores 35%, versus Uganda (70%) and Nigeria (78%), reflecting a lack of structured planning for public health threats [25]. With only 20% capacity, Somalia lags behind Ghana (70%) and Uganda (68%), weakening public awareness and response efforts [25]. Table 2 shows the key challenges to achieving IHR core capacities at PoEs in Somalia.

**Table 2:** Key challenges to achieving IHR core capacities at PoEs in Somalia

Challenge	Impact	References
Political instability	Disrupted surveillance; delayed responses; understaffed PoEs	[54]
Inadequate funding	Medical supply shortages; staff attrition; weak disease surveillance	[32]
Inadequate infrastructure	Delayed treatment; unchecked disease spread; poor rural access	[32, 106]
Decentralized management	Fragmented governance; inconsistent policies; fiscal inefficiencies	[64]

*IHR* International Health Regulations, *PoEs* points of entry

### Pathways to achieve core capacities for Somalia’s PoEs by 2025

#### Infrastructure development and compliance with IHR for PoEs

Rebuilding Somalia’s healthcare system requires restoring damaged infrastructure and improving coordination through data systems [82].Upgrading transportation networks, airports, and seaports is essential for medical logistics [83].Collaboration between government, international agencies, and local actors ensures unified efforts and informed resource allocation [84].Strengthening governance, regulation, NGO coordination, and private sector oversight is also key [72] (Table 3).

**Table 3:** Comparison of IHR core capacities at PoEs across African countries

Indicator	Somalia (%)	Kenya (%)	Uganda (%)	Ethiopia (%)	Sudan (%)	Djibouti (%)
IHR implementation capacity		80	60	75	47	27
Surveillance system capacity	30	75	60	75	60	40
Health workforce capacity	25	60	55	70	60	60
Laboratory capacity	30	80	70	75	60	27
Cross-border collaboration	25	78	68	75	50	40
Emergency preparedness	35	70	68	70	60	40
Risk communication	20	60	55	65	40	30

Capacities were scored on a 0–100% scale, with 0% indicating no capacity and 100% indicating full implementation, based on national assessments and expert evaluations

Somalia is establishing health screening facilities at PoEs and strengthening surveillance with clear indicators for monitoring [69]. Collaboration with global partners supports regulatory enforcement and health law compliance. A national PHEOC is being developed to integrate surveillance and coordinate emergency response. FETP training is enhancing public health workforce capacity. Ongoing international support remains essential to meet IHR standards [85, 86]. Deploying biometric scanners and mobile health apps for real-time IDSRS surveillance, coupled with expanded FETP training and ongoing professional development, will strengthen PoE data collection, monitoring, and workforce capacity [87].

#### Multi-sectoral collaboration, technology integration, and community engagement for PoEs

Achieving IHR core capacities at Somalia’s PoEs requires strong multi-sectoral coordination among health, customs, immigration, and transport sectors. Partnerships with WHO, UNICEF, and IOM can enhance health security and emergency response in high-risk areas [88]. Sector-specific emergency plans, integrated surveillance protocols, and regular multi-sectoral training are essential. Updating SOPs and establishing clear communication and data-sharing systems will support timely, coordinated action during health crises [89, 90].

Somalia is adopting advanced technologies at PoEs, including infrared scanners and mobile apps for health reporting and vaccination tracking [66, 91]. The Integrated Disease Surveillance and Response System (IDSRS) supports real-time data and migration intelligence for rapid public health responses [92]. Investments in telecom infrastructure aim to expand connectivity and digital services [93]. Meanwhile, IOM is strengthening border control with biometric fingerprint scanners to enhance migrant data tracking and security [94]. In addition, through the National Identification and Registration Authority (NIRA), Somalia plans to issue 15 million biometric IDs by 2026 to support governance and financial access [95].

Community engagement strengthens local response capacity through stakeholder collaboration [96]. It enables shared decision-making and builds trust and accountability [97].Partnering with local leaders enhances credibility and outreach [98].Effective strategies include using radio, social media, and leader advocacy, along with training health workers on NCD prevention and basic services [99, 100].

## Discussion

The IHR (2005) require robust surveillance, early detection, and coordinated response to manage cross-border health threats. Despite this, Somalia’s PoEs are hampered by infrastructure shortfalls, workforce gaps, and fragmented governance. This section synthesizes key deficiencies, benchmarks Somalia against regional peers, and outlines targeted strategies to strengthen its PoE systems.

Somalia’s PoEs lack essential health facilities and screening measures, with 75% of domestic airports unable to effectively prevent and control diseases during the PoE baseline assessment (June 2020) [18]. Weak disease surveillance at PoEs exacerbates health risks, leaving Somalia vulnerable to outbreaks like cholera, measles, and polio, while fragmented healthcare management limits coordination at PoEs, hindering IHR core capacity implementation, and reliance on donor aid, with only 1.3% of government health expenditure allocated domestically, creating significant funding gaps, impeding sustainable improvements [57]. While the successful PoE reforms—coordinated leadership, workforce expansion, and stronger laws—depend on stable governance, aligned federal–regional policies, targeted investment in emergency infrastructure, and international peace-building support.

Somalia’s SPAR workforce capacity sub‑score stands at around 40%, well below both the global average (~64%) and the regional average (~66%), based on the latest WHO SPAR state‑parties self‑assessment report submitted in April 2025 [19]. This gap restricts the ability of PoEs to provide essential services, particularly in rural and conflict-affected areas, where staffing is critically low [107]. Comparisons with Kenya (80% compliance) and Ethiopia (75% surveillance capacity) highlight the importance of robust legal frameworks and integrated disease monitoring, which Somalia could adopt to strengthen its systems [25, 108, 109]. To address PoE gaps in Somalia requires integrated measures: vaccination, screening, education, IPC, training, health networks, and legal protections for vulnerable groups [110]. The even stronger university and training partnerships for continuous upskilling, and leverage digital tools and mobile apps to deliver remote professional development in underserved areas.

Effective PoE upgrades require coordinated resources, including international support for medical teams and capacity-building, ongoing training for local staff, and a shift toward sustainable domestic funding through public–private partnerships and long-term donor commitments. Additionally, upgrading infrastructure such as medical screening facilities, transportation networks, and border control systems, along with increasing the availability of essential supplies like vaccines, diagnostic equipment, and personal protective equipment, is crucial. Collaboration with international partners and NGOs to rehabilitate infrastructure, particularly in conflict zones and rural areas, will ensure long-term sustainability. Investing in advanced surveillance systems, such as expanding the IDSR systems by 78% and DHIS2 by 91% in 2022, will strengthen disease monitoring [43, 44]. Real-time data collection and reporting tools should be provided to local health workers. Mobile health technologies can enhance real-time monitoring and cross-border data sharing.

As of 2023, fewer than 10% of laboratories supporting Points of Entry (PoEs) in sub-Saharan Africa were accredited or achieved SLIPTA ratings above 3 stars, and only a limited number had integrated surveillance capacities aligned with the International Health Regulations (IHR) [111]. By 2025, the goal is for ≥66% of PoE laboratories to achieve SLIPTA ≥3-star accreditation, and for ≥66% of AU Member States to have operational early-warning surveillance systems at PoEs, consistent with IHR core capacities. This will be supported through partnerships with Africa CDC, WHO, and the African Society for Laboratory Medicine, leveraging geo-mapping of laboratory networks, decentralized diagnostics, and workforce development initiative [112]. National legal reforms will be aligned with the 2024 IHR amendments, and at least one IHR-certified health inspector will be deployed at each major PoE. Workforce targets include the training and deployment of at least 30 Field Epidemiology Training Program (FETP) graduates per country—with ≥66% female representation—focusing on PoE surveillance, diagnostics, and emergency response. Furthermore, we aim to transition at least 50% of PoE health investments to domestic and private-sector co-financing, sustained through community engagement and risk communication. These efforts are part of a broader strategy to fortify regional laboratory capacity and improve pandemic preparedness [113].

Engaging local leaders and community organizations builds trust and encourages public participation in health initiatives. Educational campaigns through radio and social media can raise awareness about preventive measures, including vaccination and hygiene practices. For example, the Polio Eradication Initiative implemented by the World Health Organization (WHO) has successfully used community engagement strategies to ensure vaccination coverage in remote areas. Local leaders and community health workers play a pivotal role in mobilizing populations, addressing vaccine hesitancy, and ensuring that vaccines reach the most underserved communities. This approach has contributed significantly to the near-global eradication of polio [114]. Enhancing legislative frameworks to align with IHR standards will improve enforcement and compliance. Centralized coordination by the Federal Ministry of Health can reduce governance fragmentation and ensure consistent implementation of health regulations across regions. According to the International Health Regulation (IHR) framework, the WHO emphasizes the importance of national focal points (NFPs) within health ministries to facilitate coordination and information exchange regarding international health threats. In countries like Jamaica, for instance, the Ministry of Health & Wellness has established centralized coordination mechanisms to align their national health strategies with IHR requirements. This structure enhances compliance, improves emergency preparedness, and ensures that health measures are implemented consistently across regions [115, 116]. A Ministry of Health–led central coordination mechanism with flexible national policies harmonized to regional contexts, coupled with transparent risk communication and community engagement, will streamline PoE management. Diversifying funding, integrating private-sector and local initiatives, training local authorities, and embedding IHR into national legislation will build a resilient, accountable health emergency framework.

Somalia’s PoE vulnerabilities underscore how fragile health systems amplify global risks; fortifying these entry points and partnering with WHO and Africa CDC will bolster national security and regional stability [117, 118]. Somalia’s Points of Entry (PoE) enhancements, including the establishment of upgraded health screening facilities, improved surveillance systems, and strengthened border control measures, offer a blueprint for resource-limited countries to meet International Health Regulations (IHR) standards. These enhancements focus on improving diagnostic capabilities, ensuring timely data reporting, and enhancing cross-border collaboration. However, sustained global financial and technical support is crucial to ensure these improvements are sustainable and scalable. Future research should explore the long-term impact of these interventions on public health outcomes, as well as the effectiveness of public–private partnerships in maintaining PoE infrastructure and operations.

## Conclusions

Somalia’s progress in meeting IHR core capacities at Points of Entry (PoEs) by 2025 is critical for enhancing national resilience and global health security. The study identifies major challenges, such as inadequate infrastructure, fragmented governance structures, limited funding, and shortages in the healthcare workforce, all of which undermine the effectiveness of disease surveillance and response mechanisms. Addressing these challenges requires significant investments in PoEs infrastructure, comprehensive workforce training, and strengthening multi-sectoral collaboration. Additionally, legal frameworks must be reformed to align with international standards.

Sustainable financing, community engagement, and international support are vital to ensuring the long-term sustainability of PoE enhancements. Without these elements, the improvements at PoEs will remain short-term and may not be scalable or sustainable. Financial support is necessary to maintain and expand infrastructure; community engagement ensures local ownership and compliance with health measures, and international collaboration strengthens the capacity to respond to global health threats.

Coordinated action is urgently needed to transform PoEs into resilient health security gateways. Achieving measurable outcomes, such as a significant increase in PoE capacity to meet IHR standards and improved public health response times, will be key indicators of success by 2025.

While the study provides valuable insights into the challenges and necessary actions, it is limited by the availability of up-to-date data and regional variations in health system capabilities. The lack of comprehensive data across all PoEs and the diversity in regional health infrastructure hinder a fully accurate assessment of progress. Therefore, further research is needed to assess the long-term impact of PoE enhancements on health security and the effectiveness of cross-border health initiatives. This will help refine strategies and ensure that interventions are adapted to specific regional needs.

## Data Availability

Our correspondents are available to provide raw data at any time, ensuring the smooth functioning of our initiatives. As this is a narrative review, no primary datasets were generated. However, the authors remain available to share supporting materials or references upon reasonable request to facilitate transparency and replication.

## Declarations

### Descriptions for experiments

No experiments involving human or animal subjects were conducted. This study is based on a narrative review of published literature and publicly available data sources.
